# Evaluate the status and associated factors of *Gəˀəz* manuscripts heritage preservation: The case of *Qäranǝyo mädḫane ˁaläm* church, northwest Ethiopia

**DOI:** 10.1371/journal.pone.0345893

**Published:** 2026-08-14

**Authors:** Selomon Afework Yenesew

**Affiliations:** Department of Natural Resources Management, College of Agriculture and Environmental Sciences, Bahir Dar University, Ethiopia; Linyi University, CHINA

## Abstract

**Introduction–aims:**

Ethiopia has developed and preserved its indigenous script, which has played a significant role in shaping the country’s cultural and historical identity. Gəˀəz manuscripts represent an important component of this heritage; however, their long-term preservation is threatened by physical deterioration and environmental exposure. This study aimed to assess the preservation status and physical condition of Gəˀəz manuscripts at Qäranǝyo Mädḫane ˁAläm Church and to identify observable factors associated with their preservation status.

**Methods:**

A cross-sectional observational inventory of 71 Gəˀəz manuscripts was conducted. Data were analyzed using STATA version 17. Manuscript preservation status was classified into five ordered categories: well-preserved, better condition, good condition, bad condition, and dangerous condition. An ordinal logistic regression model was used to examine associations between observable deterioration indicators and manuscript preservation status.

**Results and interpretation:**

71 manuscripts were found at the church, comprising 13 (18.3%) from the fifteenth century, 2 (2.8%) from the sixteenth century, 42 (59.2%) from the seventeenth century, and 14 (19.7%) from the eighteenth century. Moreover, 11.3% of the manuscripts were classified as well-preserved, 26.8% as being in better condition, 31.0% in good condition, 23.9% in bad condition, and 7.0% in dangerous condition. Ordinal logistic regression showed that visible moisture damage, rodent damage, frequent handling, and insect infestation were significantly associated with poorer preservation status (P ≤ 0.001). These findings describe observable patterns and associations rather than causal relationships, highlighting the importance of routine condition assessment, improved storage environments, preventive conservation measures, and careful handling to support the long-term preservation of Gəˀəz manuscripts.

## 1. Introduction

*Gəˀəz* manuscripts are handwritten texts written in the *Gəˀəz* language. Now a days, these manuscripts are preserved in many countries and have attracted considerable interest from scholars, collectors, and cultural institutions [1]. Consequently, extensive cataloguing, digitization, and preservation initiatives have been undertaken by libraries and museums across Europe, North America, and Asia [2,3]. Major institutions, including the British Library, the Vatican Library, and the Bibliothèque nationale de France, hold substantial collections of Gəˀəz manuscripts [4]. These institutions have played an important role in digitizing and preserving manuscript collections, improving access for researchers while reducing the need to handle fragile originals and thereby helping to limit physical deterioration [5].

Ethiopia, however, remains the principal repository of Gəˀəz manuscripts. Unlike many African countries that adopted Latin or Arabic scripts [6], Ethiopia has maintained and developed its indigenous writing system, Gəˀəz (Ethiopic), which continues to play a central role in the country’s cultural, historical, and religious traditions [7]. The Ethiopian Orthodox Tewahedo Church is the principal custodian of these manuscripts, most of which are preserved in churches and monasteries [4]. Collaborative initiatives, such as the Ethiopian Manuscript Microfilm Library (EMML), have made substantial contributions to the digitization of thousands of manuscripts, thereby expanding access to Ethiopia’s written heritage [8]. Nevertheless, these initiatives have largely focused on documentation and accessibility, while comparatively little attention has been given to the physical preservation of manuscripts within their original ecclesiastical settings.

Recent advances in integrated digital technologies, including remote sensing, multispectral imaging, and advanced visualization techniques, have enhanced the documentation, monitoring, conservation, restoration, and promotion of cultural heritage [9]. However, digitization primarily preserves manuscript content rather than the physical objects themselves. It does not address the condition of manuscript materials, storage environments, or the cumulative effects of routine liturgical use and handling. Likewise, traditional storage practices, handling methods, and the religious functions of manuscripts remain integral to their preservation but have not been systematically evaluated in many Ethiopian church collections. A better understanding of these contextual factors is therefore important for developing sustainable conservation strategies that complement digital preservation initiatives [10].

Previous studies have primarily emphasized cataloguing, textual analysis, philological research, and historical description [11–16]. In contrast, this study focuses on the physical preservation status of manuscripts by combining systematic condition assessment with statistical analysis of observable deterioration indicators. Qäranǝyo Mädḫane ˁAläm Church houses a collection of 71 Gəˀəz manuscripts, of which 58 have been digitized through the Endangered Archives Programme (EAP) and 13 have been catalogued [17]. Despite these documentation efforts, no systematic assessment has been conducted to evaluate their current preservation status or to examine observable deterioration factors within the collection. Accordingly, this study provides site-specific empirical evidence by documenting the preservation condition of the manuscripts and examining associations between visible deterioration indicators and preservation status. Specifically, the study aims to (1) assess the preservation status of Gəˀəz manuscripts; (2) identify observable deterioration factors affecting their physical condition; (3) evaluate the associations between deterioration indicators and overall preservation status; and (4) provide baseline empirical evidence to support future conservation planning. The emphasis from descriptive documentation to a systematic assessment of manuscript preservation at the church level, the study addresses an important gap in Ethiopian Orthodox manuscript heritage research and provides context-specific evidence to inform future conservation planning.

## 2. Materials and methods

### 2.1. Description of the study area and manuscript assessment

**Fig 1:**
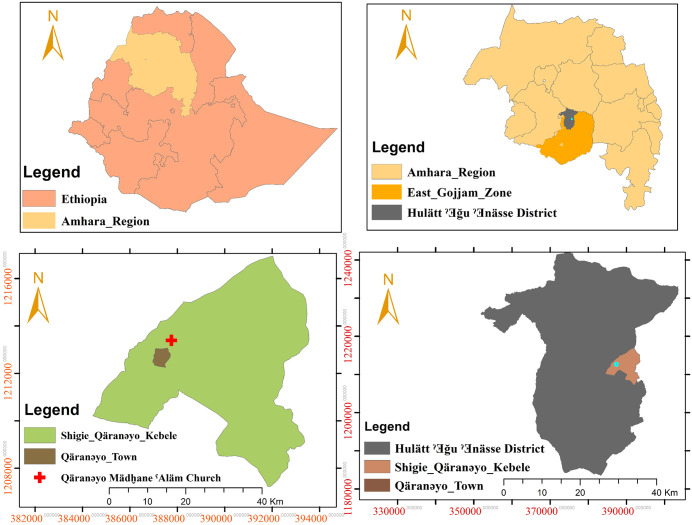
Location Map of the Study Area (Source author QGIS preparation).

This research was carried out at *Qäranǝyo Mädḫane ˁaläm Church*, located in Hulätt ˀƎğu ˀƎnässe district, East Goğğam Administrative Zone, Amhara National Regional State, Northwest Ethiopia. Geographically, *Qäranǝyo* town lies within 10° 58’ 8.72” N latitude and 37° 58’ 13.85” E longitude (Fig 1).

*Qäranǝyo Mädḫane ˁAläm* Church is one of the most ancient monasteries in the historical district of East *Goğğam* Administrative Zone. According to traditional accounts and historical records, the monastery was established in 333 A.D. by Emperors *ˀAbǝrha* and *ˀAṣǝbǝḥa*, adjacent to the Monastery of *Märtulämarǝyam* [10]. The church is historically and religiously significant, having served as a center of ecclesiastical authority. Its location, between the *Täme* and *Ṭäji ˀsǝṭǝl* rivers, is traditionally linked to the site of Calvary in Jerusalem, with the underground area of *Dǝb Gašša* considered analogous to Golgotha. This historical and spiritual significance has made the church a repository of rare and ancient manuscripts, many of which were produced or preserved during periods of reconstruction, under Emperor *Sälomon* [18].

The church also hosts traditional schools, including Singing *bǝtǝ*, *Qǝddase betǝ*, and *Qǝne betǝ*, which serve as centers for the oral transmission of ecclesiastical education, calligraphy, poetry, biblical commentary, and other forms of religious learning. This long-standing scholarly tradition has contributed to the accumulation and preservation of manuscripts over centuries.

71 *Gəˀəz* manuscripts were found in the church. Many are written on parchment, with some featuring illuminations, marginal notes, or decorative bindings, reflecting their cultural and scholarly importance. Manuscripts placed in dedicated museum-like storage areas and those in ritual use frequently show signs of wear, highlighting the challenges of preservation in both archival and functional contexts.

### 2.2. Research approach and design

This study employed a mixed-methods research design, integrating both qualitative and quantitative approaches to ensure a comprehensive and systematic assessment of Gəˁəz manuscripts. The quantitative component focused on a full-scale evaluation of manuscript preservation conditions and the identification of the factors contributing to deterioration, while the qualitative component provided an in-depth interpretive analysis of their historical, cultural, and religious significance. Document analysis involves skimming, reading, and interpreting documents [19]. Documents contain text or words and images that have been recorded without a researcher’s intervention. Manuscripts are documents that containing diverse information that requires analysis to extract relevant content [20].

Building on this methodological foundation, an in-depth investigation was conducted across the entire corpus of 71 Gəˁəz manuscripts from Qäranǝyo Mädḫane ˁAläm Church. The inclusion of the full collection, rather than a sampled subset, ensured a complete, internally consistent, and representative assessment of preservation conditions. This comprehensive dataset enabled a systematic evaluation of interacting deterioration factors, including moisture exposure, pest infestation, rodent damage, and intensive manuscript use, alongside patterns of handling and long-term preservation of the manuscripts. The study provides a robust analytical framework for understanding how these interrelated factors collectively shape manuscript status and long-term preservation outcomes.

### 2.3. Sampling design of the manuscripts

The study area was selected through a set of predefined and theoretically grounded criteria, namely the historical and religious significance of the church, the presence of a substantial and well-documented manuscript collection, and the practical accessibility of the site for systematic scholarly investigation. In line with these considerations, a purposive sampling strategy was adopted.

**Fig 2:**
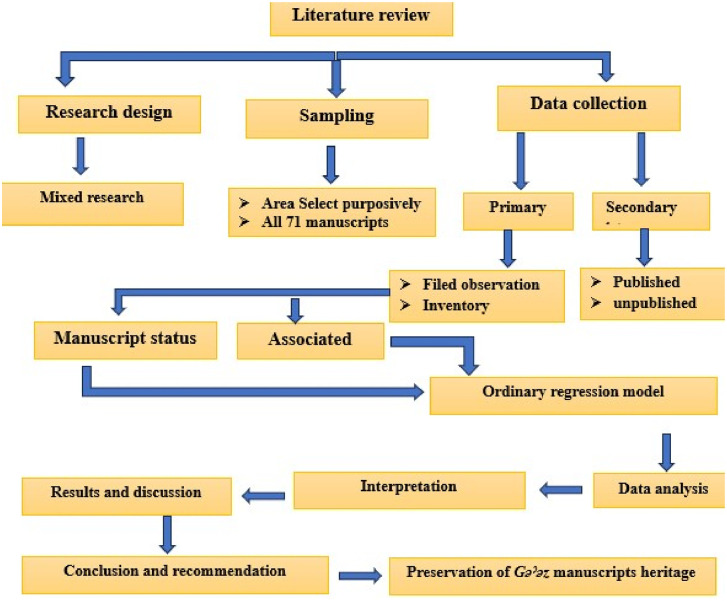
Flowchart of the research steps.

Following site selection, the entire manuscript corpus held by the church was subjected to systematic examination. The collection comprised 71 digitized and catalogued manuscripts, and instead of drawing a limited subset, a complete census of all available manuscripts was undertaken (Fig 2). This full inclusion strengthened the analytical robustness of the study by ensuring that no potentially relevant material evidence was excluded, thereby enhancing the comprehensiveness and internal validity of the assessment of the collection as a whole.

Within this complete corpus, analytical emphasis was directed toward manuscripts exhibiting visible physical deterioration, documented historical importance, and variation in material composition, script style, and binding techniques.

### 2.4. Methods of data collection

Both primary and secondary data were employed in this study to ensure a comprehensive and analytically grounded assessment of manuscript preservation conditions. Primary data were collected through systematic field observation and manuscript inventory analysis [21], with particular emphasis on a clearly defined set of preservation criteria. These criteria included the physical age of the manuscripts, binding techniques and their structural integrity, the type and condition of protective coverings and bags, and observable forms of physical deterioration. This graded deterioration framework formed the analytical basis for classifying manuscript conditions into five distinct preservation categories, reflecting progressive levels of physical decay.

In addition, secondary data sources including published books, peer-reviewed journal articles, and prior scholarly studies were used to contextualize the empirical findings and strengthen the interpretation of preservation patterns and factor affecting the preservation of the manuscripts.

### 2.5. Indicators and criteria for evaluating manuscript preservation status

**Fig 3:**
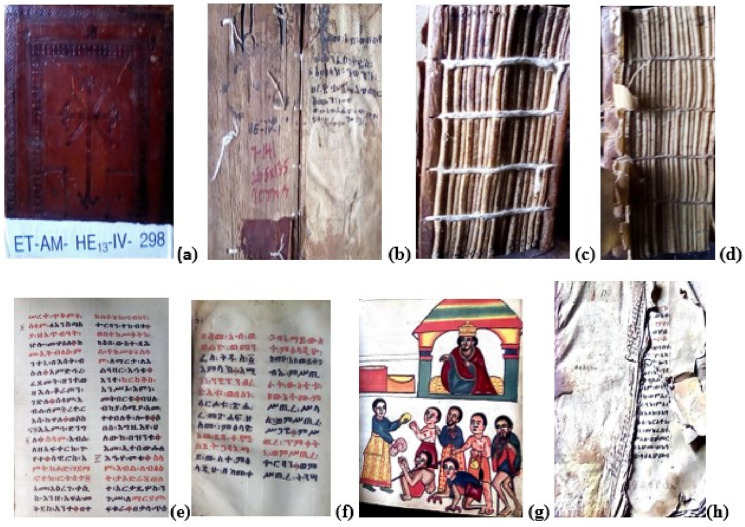
Condition status of manuscripts showing variations in preservation states: (a) well-preserved cover; (b) broken cover; (c) intact and well-preserved sewing (binding) at the spine; (d) detached or separated binding structure; (e) well-preserved text; (f) clearly readable text with visible signs of deterioration; (g) well-preserved illustrative images; (h) severely deteriorated and illegible text. (Source author data collection April, 2024).

The assessment of preservation status was based on a continuum of material degradation, ranging from manuscripts with fully legible text and illustrations and intact bindings, through intermediate stages characterized by ink fading, material brittleness, minor tears, and early signs of binding stress, to more advanced deterioration involving parchment separation, loss of manuscript sections, and extensive damage caused by insects, moisture, and mildew (Fig 3). At the most severe end of this continuum, manuscripts exhibit extreme structural disintegration, where the material becomes highly fragile and textual content is largely compromised or lost [1,17]. The statuses of the manuscripts were classified into five categories: well-preserved better condition, good condition, bad condition and dangerous condition. The detail of each condition presented Table 1.

**Table 1:** Criteria used to identify the preservation status of the manuscript.

Category	Description	Typical Features / Damage
Well-Preserved	Minimal signs of deterioration; parchment remains supple, writing and illustrations are clear; binding intact.	Parchment supple and stableText and illustrations fully legibleBinding unaffected
Better Condition	Slight deterioration, but text highly legible; overall integrity not affected.	Minor wear on edgesText remains clearSmall blemishes or slight discolorationBinding mostly intact
Good Condition	Visible deterioration, but manuscript largely intact and readable.	Moderate fading of inkSlight parchment brittlenessSmall tears at edgesMinor binding issues
Bad Condition	Manuscripts heavily used; parchment separation, ink faded, and text partially difficult to read.	Parchment separated from bindingCracks and significant ink fadingLarge tears or missing sectionsInsect, moisture, or mildew damage
Dangerous Condition	Manuscripts severely damaged; extremely fragile; partial or total loss of text.	Extensive damage from insects, rats, moisture, or mildewParchment disintegrating at touchInk smudged or barely visibleBinding completely compromised

Sources [1,17].

### 2.6. Ordinal regression model specification

An ordinal regression model was employed to analyze the factors influencing manuscript preservation. In this model, the relationship between independent variables (associated factors) and the ordinal dependent variable (preservation status) is examined through coefficients. These coefficients indicate both the strength and direction of each factor’s influence on the preservation status. Positive coefficients suggest a higher likelihood of preservation in better conditions as the factor increases, while negative coefficients suggest an association with poorer preservation. The general equation for an ordinal regression model, as provided by Liu and Koirala [22], can be expressed as follows:


$$\begin{array}{c} \text{log}\left(\frac{P\left(y\leq j|\cdot X\right)}{P\left(y>j|\cdot X\right)}\right)=\beta_{o}^{j}+\beta_{\text{1}\;}\;X_{\text{1}}+\;\beta_{\text{2}}X_{\text{2}\;+\;\cdots \;+\;\beta_{k\;}\;X_{k}} \end{array}$$

Where:

P(y≤j|·X) The probable status of manuscript preservation is less than or equal to category j given the prediction X.

P(y>j|·X) The probable status of manuscript preservation is greater than category j

X represents the vector of independent variables (associated factors affecting preservation).

$\beta_{o}^{j}$ The threshold parameter of category j

$\beta_{\text{1}\;},\;\beta_{\text{2}\;.\;.\;.\;\beta_{k}}$ The coefficient for predict variable $X_{\text{1}},X_{\text{2}}{\;.\;.\;.\;X}_{k}$ factor include age, cover, bag, moisture, termites, rat and intensive use of manuscripts.

The dependent variable was manuscript preservation status, coded as an ordinal outcome with five ordered categories: 1 = Well Preserved, 2 = Better Condition, 3 = Good Condition, 4 = Bad Condition, and 5 = Dangerous Condition, where higher values indicate progressively poorer preservation status (Table 2).

**Table 2:** Coding of the dependent variable.

Variable	Description	Coding (Ordered Categories)
Preservation Status	The preservation status of the manuscript (Ordinal outcome)	1 = Well Preserved (Best condition) < 2 = Better Condition < 3 = Good Condition < 4 = Bad Condition < 5 = Dangerous Condition (Worst condition)

The independent variables included manuscript age (15th, 16th, 17th, and 18th centuries), availability of a manuscript cover, availability of a protective storage bag, and deterioration factors recorded as separate binary variables: moisture damage, termite damage, rat damage, and deterioration due to intensive use (Table 3).

**Table 3:** Coding of the independent variables.

Variables	Description	Value Labels	Expected Sign
Age	Century of the manuscript	1 = 15th Century; 2 = 16th Century; 3 = 17th Century; 4 = 18th Century	±
Cover	Availability of manuscript cover	0 = No; 1 = Yes	+
Bag	Availability of protective storage bag	0 = No; 1 = Yes	+
Moisture	Presence of moisture damage	0 = No; 1 = Yes	−
Termite	Presence of termite damage	0 = No; 1 = Yes	−
Rat	Presence of rat damage	0 = No; 1 = Yes	−
Intensive Use	Presence of deterioration due to intensive use	0 = No; 1 = Yes	−

The proportional odds assumption was evaluated using the Test of Parallel Lines. Model adequacy was assessed using the Pearson and Deviance goodness-of-fit tests, while overall model significance was evaluated using the likelihood ratio test comparing the intercept-only and final models. The contribution of each predictor was assessed using likelihood ratio tests and Wald chi-square statistics. Adjusted odds ratios (AORs) with 95% confidence intervals (CIs) were estimated to quantify the strength and direction of associations between explanatory variables and manuscript preservation status.

### 2.7. Data analysis

Data collected from fieldwork and manuscript inventory were processed and analyzed using STATA Version 17, involving data cleaning, coding, categorization, and statistical analysis. Python 3.14 was used for graphical visualization of results. The analysis began with descriptive statistics, including frequencies and percentages, to summarize the distribution of manuscript characteristics and preservation status. In addition to descriptive analysis, inferential statistical techniques were applied to determine the relationship between explanatory variables and manuscript preservation status. The strength and direction of associations were evaluated using regression coefficients (B) and their transformation into adjusted odds ratios (AORs). Statistical significance was assessed using Wald chi-square tests and 95% confidence intervals (CI) at a significance level of *p* < 0.05.

### 2.8. Model fit and goodness-of-fit results

The model fit and goodness-of-fit statistics indicate that the ordinal logistic regression model provides an overall acceptable representation of the data. The Pearson and Deviance goodness-of-fit tests were not statistically significant, suggesting that there is no strong evidence of lack of fit and that the model adequately describes the observed data (Table 4).

**Table 4:** Goodness-of-Fit Statistics (Ordinal Logistic Regression).

Test	Chi-Square	df	Sig. (p-value)
Pearson	62.48	64	0.55
Deviance	58.91	64	0.66

In the assessment of individual predictors using chi-square tests, manuscript age and cover availability were not significantly associated with manuscript preservation status (Table 5). However, the availability of a storage bag and deterioration signs showed statistically significant associations, indicating that these factors are important contributors to variations in manuscript condition.

**Table 5:** Chi-Square test statistics.

Variable	Pearson Chi-Square	df	p-value
Age (Century)	11.76	12	0.46
Cover	1.98	4	0.74
Bag	11.52	4	0.021
Deterioration Sign	39.84	16	0.001

The model fitting information further confirms the overall usefulness of the regression model. The final model shows a substantial improvement over the intercept-only model, indicating that the included predictors collectively provide a significantly better explanation of manuscript preservation status (Table 6).

**Table 6:** Model Fitting Information (Ordinal Logistic Regression).

Model	−2 Log Likelihood	Chi-Square	df	Sig. (p-value)
Intercept Only	142.86	—	—	—
Final Model	93.42	49.44	9	<0.001

## 3. Results

### 3.1. Preservation status and age the manuscripts

**Fig 4:**
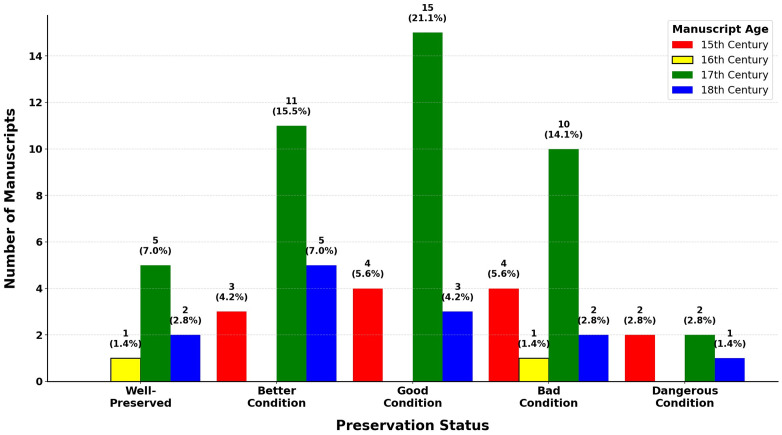
Perseveration status of manuscript with age distribution.

The analysis of the preservation status and chronological distribution of the manuscripts, as illustrated in Fig 4, provides a clearer understanding of both their historical development and long-term survival patterns. The preservation status of the 71 manuscripts reveals a complex pattern that goes beyond a simple classification of conditions and instead reflects interacting historical, material, and environmental processes shaping manuscript survival. The distribution shows that only 11.3% of the manuscripts are well preserved, while 26.8% are in better condition and 31.0% are in good condition. Together, these relatively stable categories account for just under 70% of the collection. However, a substantial proportion remains at risk, with 23.9% classified as in bad condition and 7.0% in dangerous condition, indicating that nearly one-third of the manuscripts are experiencing significant deterioration.

The manuscripts in the collection span from the 15^th^ to the 18^th^ century, reflecting sustained manuscript production over several centuries within the church tradition. The quantitative distribution shows a highly uneven temporal pattern: 59.2% of the manuscripts are attributed to the 17^th^ century, 29.7% to the 18^th^ century, 18.3% to the 15^th^ century, and only 2.8% to the 16^th^ century. The dominance of the 17^th^–18^th^ century manuscripts (78.9%) (Fig 4) is analytically significant, as it reflects not simply production intensity but also differential survival and preservation probability across time.

### 3.2. Binding (cover) material of the manuscripts

The analysis of manuscript preservation in relation to binding materials provided in Table 7. Various binding materials Wooden Board (WB), Decorated Leather (DL), Half Leather (HL), Hard Skin (HS), and No Cover (NS) demonstrate differing impacts on the condition of the manuscripts. The majority of manuscripts (40.8%) bound in DL and WB. DL bounded manuscripts found in better condition. Five manuscripts are classified as being in a dangerous condition bound using WB, indicating a critical preservation state.

**Table 7:** Binding (cover) material of the manuscripts.

Preservation /Condition	Binding Material (Cover)						Total	%
	WB + DL + C	DL	HL	HS	WD	NS		
Well-Preserved	1	3		2	2		8	11.3
Better Condition	2	11	2		4		19	26.8
Good Condition	1	8	3		9	1	22	31.0
Bad Condition	1	7			9		17	23.9
Dangerous Condition					5		5	7.0
Total	5	29	5	2	29	1	71	100
%	7.0	40.8	7.0	2.8	40.8	1.4	100	

WB = Wooden Board; DL = Decorated Leather; C = Cloth; HL; Half Leather; HS = Hard Skin; NC = No Cover.

(Source author data collection April, 2024).

### 3.3. Bags of the manuscripts

**Fig 5:**
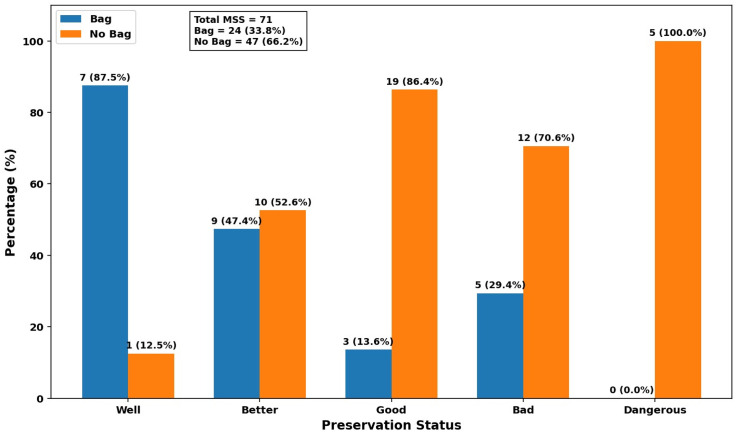
Relationship between protective storage (bag vs. no bag) and preservation status of Gəˁəz manuscripts.

The relationship between protective bags (*näṭäla maḫədär*) and manuscript preservation status demonstrates a clear association between storage practices and long-term material survival. The findings reveal a distinct preservation gradient shaped by the presence or absence of protective coverings, indicating that storage conditions function as a major associated factor of manuscript durability alongside factors such as age, handling history, and material composition. Of the 71 manuscripts examined, only 24 (33.8%) were preserved with protective leather bags, while the majority 47 manuscripts (66.2%) lacked such coverings, exposing them to prolonged environmental and mechanical stress. The findings indicate that manuscripts stored in protective bags generally exhibited better preservation conditions than those kept without protective coverings. Among the eight manuscripts classified as well-preserved, seven manuscripts (87.5%) were stored in bags, while only one manuscript (12.5%) lacked protective storage (Fig 5). These manuscripts retained intact covers, stable bindings, clearly legible texts, and minimal signs of environmental or biological deterioration. This suggests that protective storage bags play a significant role in minimizing exposure to dust, moisture fluctuation, insect infestation, and mechanical damage.

### 3.4. Factor affecting the status of the manuscripts

Based on the analysis, four major factors were identified as contributing to the current condition of the manuscripts: moisture, rat damage, intensive use, and insect infestation. As shown in Table 8, these factors affect the manuscripts at varying rates. Moisture is the most widespread stressor, affecting 57.8% of manuscripts; rat damage accounts for 15.5%; intensive use contributes to 14.1%; and insect infestation affects 7.0% of manuscripts. Additionally, 5.6% of the manuscripts show no visible signs of deterioration, indicating relatively good preservation. These stressors affect various parts of the manuscripts, including the binding materials, folios (pages), and sewing threads.

**Table 8:** Factor affecting the status of the manuscripts.

Preservation /Condition	Factor					Total	%
	No	Moisture	Insect	Intensively use	Rat		
Well-Preserved	4	4				**8**	**11.3**
Better Condition		16	1		2	**19**	**26.8**
Good Condition		15		4	3	**22**	**31.0**
Bad Condition		4	3	6	4	**17**	**23.9**
Dangerous Condition		2	1		2	**5**	**7.0**
Total	4	41	5	10	11	**71**	**100**
%	5.6	57.8	7.0	14.1	15.5	**100**	

(Source author data collection April, 2024).

### 3.5. Factors associated with manuscript preservation status

The ordinal logistic regression results show clear differences in how the independent variables influence manuscript preservation status, based on the direction of the regression coefficients (B) and statistical significance levels. The results indicate that manuscript age is not statistically significant (*p* > 0.466). Although the coefficient is positive, the lack of significance means that there is no strong evidence that manuscripts from different centuries differ in their preservation status (Table 9). In other words, older or newer manuscripts in this dataset do not show a systematic difference in deterioration level. Similarly, cover availability is also not statistically significant (*p* > 0.706). The negative coefficient suggests a potential protective tendency, but since it is not statistically significant, this effect is not reliable. Therefore, the presence or absence of a cover does not meaningfully explain variation in manuscript preservation status in this model. In contrast, the availability of a storage bag is statistically significant (*p* < 0.001) (Table 9). The negative coefficient indicates that manuscripts stored in bags are associated with lower odds of being in worse preservation categories. This suggests a protective role of storage bags in reducing deterioration and improving manuscript preservation outcomes.

**Table 9:** Variable estimates of manuscript preservation associated factors.

Variable	Estimate (B)	Std. Error	Wald	df	Sig. (p-value)	95% CI Lower	95% CI Upper
Age	0.31	0.42	0.54	1	0.466	−0.51	1.13
Cover	−0.44	0.34	0.14	1	0.706	−1.10	0.22
Bag	−0.82	0.24	11.12	1	<0.001	−1.29	−0.35
Moisture	−1.25	0.30	17.36	1	<0.001	−1.84	−0.66
Insect	−2.98	0.88	11.45	1	<0.001	−4.70	−1.26
Intensive use	−2.10	0.65	10.42	1	<0.001	−3.37	−0.83
Rat	−1.76	0.59	8.89	1	<0.001	−2.92	−0.60

All deterioration factors show strong and statistically significant relationships with manuscript preservation status (p < 0.001). Moisture damage indicates that manuscripts affected by moisture are significantly more likely to be in poorer preservation conditions. Insect damage shows the strongest effect among all variables, indicating a very strong association with severe deterioration. Intensive use also has a significant negative effect, suggesting that frequent handling or use significantly contributes to manuscript degradation. Similarly, rat damage is significantly associated with worse preservation status, indicating that biological damage from rodents contributes substantially to deterioration.

## 4. Discussion

### 4.1. Preservation conditions with age distribution

The dating of the manuscripts was established through a rigorous synthesis of both internal and external lines of evidence rather than reliance on a single indicator. Primary sources of dating information included colophons, donation records, ownership notes, marginal annotations, and contextual evidence preserved on bindings, covers, and protective bags. Most of the manuscripts were written during 17^th^ and 18^th^ century. The age distribution of the manuscripts reflects significant historical transformations that shaped the survival and reproduction of Ethiopian manuscript culture [23]. A major explanatory factor is the widespread destruction associated with the invasion of *Gragn Ahmed*, during which numerous churches, monasteries, and manuscript repositories were burned or looted [24,25]. The comparatively limited survival of earlier manuscripts therefore reflects not only natural deterioration but also the large-scale loss of documentary heritage during this period of conflict. On the other hand, the dominance of manuscripts from the 17^th^ and 18^th^ centuries corresponds to a subsequent phase of ecclesiastical and intellectual reconstruction. During this recovery period, church institutions intensified manuscript copying and reproduction activities in order to rebuild liturgical, theological, and scholarly collections that had been destroyed. This can be attributed to the reconstruction of the church following the fire damage caused by *Gragh Ahimed*. *Ras Märǝd* and King *Sälomon* played vital roles in preserving various manuscripts encompass religious, philosophical, historical, social law, statecraft, cultural, mathematical, astronomical, and other related disciplines [1,20].

From a preservation perspective, the age distribution of the manuscripts also reveals a clear survival pattern shaped by long-term exposure to deterioration processes. The relatively larger proportion of 17^th^ - and 18^th^ -century manuscripts can partly be explained by their shorter exposure to environmental and biological degradation, including ink fading, parchment weakening, pest infestation, and cumulative handling damage. Conversely, the scarcity of earlier manuscripts reflects both historical destruction and progressive material deterioration over extended periods. This uneven survival pattern demonstrates that manuscript preservation is influenced not only by historical production rates but also by differential rates of survival through time [26].

**Fig 6:**
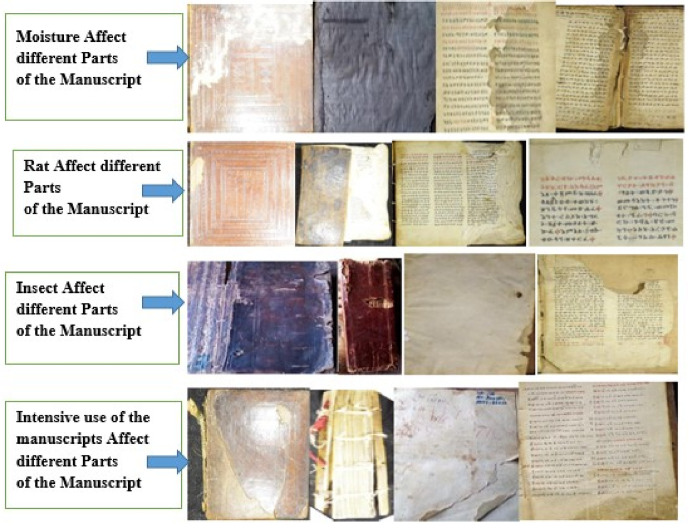
Sample, factor affecting the status of the manuscripts (Source author data collection April, 2024).

Manuscripts classified as well-preserved, many of which originate from the 16^th^ and 18^th^ centuries, indicate that preservation outcomes are not determined exclusively by age. Instead, these manuscripts appear to have benefited from relatively stable storage environments, limited handling, and, in some cases, informal conservation practices. The largest preservation category consists of manuscripts classified as being in good condition, particularly those dating from the 17^th^ century. These manuscripts generally remain structurally intact but already exhibit early indicators of deterioration, including fading ink, weakened bindings, minor biological damage, and surface abrasion (Figs 3, 6).

On the other hand, manuscripts categorized as being in bad or dangerous condition represent a major conservation concern. These manuscripts are associated with older collections, particularly those originating from the 15^th^ and 17^th^ centuries. Their condition reflects the cumulative effects of prolonged exposure to humidity, biological infestation, repeated handling, and unstable storage environments. The concentration of severe deterioration among older manuscripts also indicates a broader survival bias, whereby only the most vulnerable remnants of earlier manuscript traditions remain, while many others may already have disappeared entirely through irreversible degradation or historical destruction [27].

### 4.2. Preservation conditions with protective material

Protective materials and storage systems also emerged as important associated factor of manuscript survival. Manuscripts lacking protective coverings were consistently more vulnerable to environmental and physical damage, highlighting the importance of proper conservation support. Previous studies indicate that combinations of wooden boards, leather bindings, and cloth coverings provide enhanced structural protection and durability [28]. Leather, in particular, was widely used because of its flexibility and resilience, although it remains susceptible to degradation under fluctuating environmental conditions [29–31]. Shelley [32] similarly argues that poor preservation outcomes are often associated not only with binding weakness but also with inadequate storage systems and unstable environmental conditions that accelerate material deterioration.

A relatively balanced distribution was observed among manuscripts categorized as being in better condition, where some manuscripts were preserved in protective bags while others remained unprotected. Although these manuscripts generally retained structural functionality and textual readability, many already displayed visible signs of deterioration (Figs 3, 6). Manuscripts classified as being in bad condition were predominantly stored without protective bags and exhibited detached bindings, torn folios, biological damage, and increasing structural fragility. The most severe deterioration was observed among manuscripts categorized as being in dangerous condition, all of which lacked protective storage. These manuscripts displayed advanced stages of degradation, including illegible texts, severe biological infestation, detached components, and extensive physical disintegration (Figs 3h, 6).

### 4.3. Factor associated with manuscript preservation status

The analysis identified four principal factors contributing to the current preservation condition of the manuscripts: moisture, rat damage, intensive use, and insect infestation (Fig 6). These deterioration agents affect the manuscripts at different levels of severity and frequency. Among them, moisture emerged as the most pervasive threat, affecting more than half of the surveyed manuscripts. Rat damage and intensive use also represent significant sources of deterioration, while insect infestation affects a comparatively smaller but still important proportion of the collection. Only a limited number of manuscripts showed no visible signs of deterioration, indicating that relatively few manuscripts remain in stable preservation condition.

Moisture constitutes the most destructive environmental factor affecting manuscript preservation. Its impact extends across both external and internal manuscript components, compromising parchment quality, weakening bindings, deforming folios, and accelerating ink deterioration. Prolonged exposure to humid conditions frequently results in mold growth, parchment softening, discoloration, and fading of written text [33]. In many cases, moisture also creates favorable conditions for secondary biological damage, particularly fungal and microbial activity, which further accelerates material decay [34]. The widespread occurrence of moisture-related deterioration highlights the absence of adequate environmental control systems within many storage environments and demonstrates the critical importance of humidity regulation for long-term manuscript preservation [35]. Previous research indicates that high moisture levels accelerate microbial growth and organic decay, particularly affecting cellulose and other plant-based materials commonly used in manuscript production [36].

Rat infestation represents another major preservation challenge, particularly among manuscripts already categorized as being in poor or dangerous condition. Rodents commonly gnaw on bindings, folios, and sewing structures, producing severe physical damage that is often irreversible [37]. In addition to direct destruction, rat activity contributes to further undermining manuscript integrity (Fig 6). The concentration of rodent damage among already deteriorated manuscripts is especially alarming because these collections are at heightened risk of complete structural collapse and potential loss of textual content [38]. This pattern indicates that inadequate storage protection and poor repository management significantly increase vulnerability to biological threats [39,40].

Intensive use also emerged as a significant factor influencing manuscript deterioration, particularly for manuscripts regularly used in liturgical and religious practices. Manuscripts classified as being in bad condition commonly display visible signs of wear associated with repeated use, including damaged folios, detached bindings, and surface erosion (Fig 6). Frequent handling exposes manuscripts to continuous mechanical stress, leading to abrasion, tearing, weakening of sewing structures, and gradual breakdown of binding materials [17]. The findings therefore illustrate the preservation challenges created by the tension between continued functional use and long-term conservation.

Insect infestation constitutes an additional biological threat, particularly among manuscripts already categorized as being in bad or dangerous condition. As illustrated Fig 6 Insects commonly attack both external and internal manuscript components, including leather bindings, sewing threads, wooden boards, and parchment folios [41]. Pests such as termites and bookworms consume organic materials, progressively weakening the structural cohesion of manuscripts and increasing their fragility over time [36]. The presence of insect-related deterioration further demonstrates the vulnerability of manuscript collections stored in environments lacking effective pest management and routine preservation monitoring [42].

### 4.4. Strengths and limitations

This study provides an inclusive and empirically grounded assessment of Ethiopian Gəˁəz manuscript preservation by integrating multiple sources of evidence, including field observation and systematic inventory analysis. The use of clearly defined classification criteria covering age, binding condition, protective materials, and visible deterioration ensures a structured and transparent evaluation of preservation status. The application of statistical analysis further strengthens the analytical rigor and clarity of presentation. In addition, the study adopts a multidimensional framework that captures environmental, biological, and human factors simultaneously, allowing for a more holistic understanding of manuscript deterioration processes. This contributes valuable empirical evidence to an under-researched area of Ethiopian manuscript heritage.

Despite its strengths, the study is limited by its focus on 71 Gəˀəz manuscripts from a single church. Preservation assessments were based on visual inspection and inventory data without laboratory analyses or continuous environmental monitoring, limiting the detection of microscopic deterioration and the evaluation of environmental influences. In addition, the cross-sectional design identifies associations rather than causal relationships or long-term deterioration trends. Future research should incorporate multi-site sampling, laboratory-based material analyses, environmental monitoring, and longitudinal assessments to provide a more comprehensive understanding of manuscript preservation.

## 5. Conclusions

The assessment of 71 historical Gəʿəz manuscripts demonstrates that their preservation condition is shaped by multiple interacting factors, including manuscript age, binding condition, protective materials, environmental exposure, and handling practices. Statistical analysis indicates significant associations between preservation status and deterioration indicators such as moisture damage, pest infestation, and frequent handling, while older manuscripts, particularly those dating to the fifteenth century, exhibit greater vulnerability than later collections. These findings underscore the importance of preventive conservation strategies and appropriate protective enclosures, improved binding maintenance, and regulated access and handling. Strengthening these measures through improve environmental management, appropriate protective measures, careful handling practices, and systematic monitoring to support evidence-based conservation planning and the long-term preservation of Ethiopia’s Gəʿəz manuscript heritage.

## 6. Recommendations

Based on these findings, the following targeted recommendations are proposed: (1) systematic digitization of all manuscripts to reduce physical handling and ensure long-term digital preservation; (2) implementation of regular training programs for clergy and custodians on basic conservation practices and proper manuscript handling; (3) improvement of storage conditions through environmental control measures such as humidity regulation, ventilation, and pest management systems; and (4) strengthening collaboration between local churches, national heritage institutions, and international conservation programs to provide technical, financial, and institutional support. These interventions are essential to ensure durable preservation, minimize deterioration risks, and safeguard the cultural and historical value of Ethiopia’s manuscript heritage for future generations.
